# Supplementary material for: How far can air pollution affect tourism in China? Evidence from panel unconditional quantile regressions
Source: PLoS One. 2024 Jun 7;19(6):e0304315. doi: 10.1371/journal.pone.0304315 (PMC11161063; doi:10.1371/journal.pone.0304315)
Supplement: Attachment — Submitted filename: Response to Reviewers.docx [file pone.0304315.s002.docx]

Reviewer #1: How Much Can Air Pollution Influence Tourism? Examination of 284 Chinese Prefecture-level Cities

Reviewer report:

Congratulations on the comprehensive work presented in this manuscript. However, before this can make it to press, I have several questions and suggestions that require clarification:

1. How was the weighting system for tourism resources (e.g., world cultural heritage, national scenic spots) determined, and what was the rationale behind assigning specific weights to each category?

**[Our response]** Thank you for the question. Our research seeks to explore the correlation between tourism revenue, tourist visits, and air pollution in China at the city level, while such correlation may be mediated by the importance of tourism resources. To examine the above issue, it is necessary to quantify and distinguish the importance of various tourism resources. Therefore, we employed the Chinese “Classification and Evaluation of Quality Rating of Tourist Attractions” [GB/T17775-2003] and pertinent literature to do so. We assigned the following weights to determine the relative importance of tourism resources: 3 for world cultural and natural heritage sites, 2 for 5A scenic spots, and 1 for national scenic spots, nature reserves, and forest parks. We have added the above information to Section 3.1.3. Control variables in the revised manuscript.

2. Given that there are multiple sources to obtain PM2.5 concentration data, such as direct information from China’s environmental bureau and the LandScan method, how do you ensure consistency and accuracy in the PM2.5 measurements used in this study?

**[Our response]** Thank you for the question. In our study, we prioritised using raster data for measuring PM_2.5_ concentration owing to the uniformity, multi-temporal capacity, and extensive coverage of the raster data. The raster PM_2.5_ data employed in this study are retrieved from Donkelaar et al. (2021), which are highly accurate satellite observations augmented with measurements from ground-level observation stations. As per van Donkelaar et al. (2021), the measurements of PM_2.5_ across mainland China are retrieved from http://beijingair.sinaapp.com/, which are originally extracted by individuals from live data provided by the China Environmental Protection Agency’s website. Hence, the data about China are already effectively calibrated with official ground-level observation data. Besides, the associated research paper by Donkelaar et al. (2021) has been cited more than 200 times on Google Scholar, with numerous citations about Chinese studies. This may underscore the credibility of the PM_2.5_ data employed in this study.

3. The study references the use of the artificial impervious area dataset by Gong et al. (2020) for specifying the spatial coverage of urban areas. How does this dataset compare with other available methods, such as the GHS build-up grids, in terms of capturing the true essence of urban built-up areas in China?

**[Our response]** Thank you for the question. In our previous studies, we employed the GHS build-up data to measure changes in urban areas. However, we have realised that GHS build-up data is limited to sporadic years, with gaps between the data time slices. Therefore, in this study, we used the artificial impervious surface data from Gong et al. (2020) to measure urban areas. The rationale behind this is that scholars such as Sabo et al. (2018) have noted that traditional global reference datasets were mainly compiled to confirm land cover classifications, often subsuming built-up regions within broader categories like “urban” or “artificial surfaces.” Furthermore, a detailed comparison reveals that the “artificial impervious layer” closely aligns with the “urban built-up area” as outlined by China’s “Standards for Basic Terminology of Urban Planning” (GB/T 50280-98), in which urban built-up area is defined as “land within a city's administrative boundaries that have been expropriated and developed for non-agricultural construction purposes.”

4. With respect to the transportation infrastructure variables, how did you handle cities that might have multiple railway stations or airports of different grades? For instance, if a city has both a third and fifth-level railway station, how is that incorporated into the model?

**[Our response]** Thank you for the question. In this study, cities with multiple transportation facilities of varying grades were incorporated into the model using the aggregated weighted score method. For cities with multiple railway stations/airports of different grades, each station/airport was assigned a weight based on its grade. For example, the railway stations in levels 9, 7, 5, 3, and 1 were assigned to the values 1 to 5, respectively.

To calculate the city’s overall transportation infrastructure score, we summed the weighted values of all the stations or airports within the city. Regarding your question, a city with both a third-level railway station and a fifth-level railway station would have a combined score of 7 [4 for the third-level station + 3 for the fifth-level station]. This approach allows us to effectively incorporate cities with multiple transportation facilities of different grades into the model, providing a comprehensive measure of the city’s transportation infrastructure. We have added the above information to Section 3.1.3. Control variables in the revised manuscript.

5. The study controls for various socioeconomic factors like GDP and the ratio of public fiscal expenditure to GDP. Are there any potential confounding variables that were not included in the model, and how might they influence the findings?

**[Our response]** Thank you for the question. Yes, factors such as weather conditions, wind speed, and Air Quality Index (AQI) could potentially influence our findings. These factors have been shown in prior studies to impact air pollution and tourism development in individual cities or smaller samples. Regrettably, acquiring such granular data is difficult in large-scale studies. Hence, we haven’t incorporated these factors into this study.

Empirical Method:
6.
• The method assumes observations are independent, which can be problematic for time-series or spatial data.

**[Our response]** Thank you for highlighting this issue. We utilised a panel dataset that merges time series with cross-sectional observations. Within our model, we have accounted for both temporal and city-specific fixed effects. The fixed effects model is designed to neutralise individual-specific heterogeneity that remains constant over time, ensuring that these idiosyncratic differences do not skew the estimated outcomes. Furthermore, this approach of controlling for fixed effects also aids in addressing potential issues related to endogeneity.

• With multiple coefficients for each quantile, the interpretation and comparison of results can be intricate.
• Have you considered simpler alternatives, such as Median Regression? This approach targets the median (or 50th quantile) of the dependent variable and offers a robust analysis without assuming specific error distributions. Not only is it less computationally intensive, but it also provides a more parsimonious view of the data, focusing on the median's relationships.
Could you kindly elaborate on your choice of QR over such alternatives and address the aforementioned concerns?

**[Our response]** Thank you for your suggestions. It’s true that some analyses solely use the median, or 50th percentile, for refined regression to contrast with the average outcomes from the OLS regressions. This approach is particularly useful as it reduces the biases that can arise from outliers, non-normal distributions, and heteroscedasticity that might skew mean regressions. However, our research focuses on how air pollution affects cities with varying tourism sector levels. Essentially, we’re looking at the nonlinear relationship between air pollution and tourism. To illustrate this complex interaction, we need to rely on multiple coefficients. We have also included some graphs that present the nonlinear changes in coefficients (Figures 1–6), making it easier for readers to grasp the evolving trends and their implications. This approach aligns with the standard methodologies employed in most papers on quantile regression.

7. Your analysis mentioned that in cities with low tourist visit numbers (TOV), higher PM2.5 is associated with an increase in TOV, which contradicts the findings of Shah et al. (2022). Could you elaborate on the potential factors or unique circumstances present in these cities that might account for this positive correlation between air pollution and tourism? Additionally, how do you reconcile this with the prevailing literature that often associates increased air pollution with a reduction in tourism?

**[Our response]** Thank you for the questions. While most research, including Shah et al. (2022), suggests a negative correlation between PM_2.5_ and tourism due to health concerns, we observe a different trend at lower quantiles (Q10) in our study. This does not contradict the mainstream literature, as our research confirms the negative relationship at Q50. However, as you employ the quantile regression (QR) method, we can find the nonlinear relationship between air pollution and tourism development.

In cities with early stages of tourism development, infrastructure development might be underdeveloped, but as infrastructure activity increases, leading to heightened air pollution, tourism development might also rise. This phenomenon is supported by our outcomes in Table 1, showing a significant positive correlation between road transport (ROAD) and tourism at Q10.

Shah et al. (2022) utilised the quantile regression approach to examine the effect of air pollution on international tourism in the BRICS nations. They proposed that democracy could adversely impact tourism development, as increased income might lead to a decline in a country’s environmental performance. Our research, while acknowledging the intricate relationship between air pollution and tourism development, examines different factors, thereby contributing to a more nuanced understanding of these interactions.

8. Given that the interaction effect between PM2.5 and TOU on tourist visits (TOV) is non-linear, how does the magnitude of this effect vary across different percentiles (Q10, Q50, and Q75) and what implications does this have for tourism development in regions with varying air pollution levels?

**[Our response]** Thank you for your question. Our study has found a surprising positive correlation between increased PM_2.5_ levels and the growth of tourist visits in areas where tourism is less developed, particularly in the 10^th^ percentile (Q10). This suggests that even amidst rising pollution during the early stages of tourism infrastructure development, the tourism sector can still thrive. It implies that initial pollution may not deter tourism growth in these regions but rather may coincide with infrastructure improvements. Policymakers are thus encouraged to strike a judicious balance between environmental stewardship and infrastructure advancement.

Our further analysis sheds light on the changing dynamics at different stages of tourism development. In the 50^th^ percentile (Q50), a higher PM_2.5_ concentration hurts tourism visitation. However, in the 75^th^ percentile (Q75), this negative influence fades, indicating that mature tourism sectors may be resilient to the pressures of air pollution. For regions in the mid-range of tourism development, prioritising air quality improvements and PM_2.5_ reduction is crucial for attracting and sustaining tourist numbers. In contrast, for highly developed tourism hubs, the immediate impact of increased PM_2.5_ may be less pronounced. Still, it remains essential to focus on long-term environmental sustainability as a key policy direction. We have added the above information to Section 5.1. Strategic development in tourism to cope with air pollution in the revised manuscript.

9. The study highlights the potential dangers of the "resource curse" in the context of tourism development. How can cities with abundant tourism resources, such as Shanxi, ensure they capitalize on their historical and cultural assets without falling victim to the pitfalls of over-reliance on a single industry.

**[Our response]** Thank you for your question. We realise that in those cities with abundant tourism resources, the associated reliance on the tourism industry may have unfavourable consequences. For example, in Sanya, Hainan, which is primarily a tourist destination, the drastic decrease in tourist numbers caused by the COVID-19 pandemic resulted in the bankruptcy of Hainan Airlines, severely affecting the local economy. To avoid the risks of over-reliance on tourism resources, we advocate, in addition to diversifying the economy, diversifying the uses of tourism resources to strengthen the tourism sector’s resilience. For example, rather than relying on tourist attractions to attract tourists, Beijing’s Palace Museum has leveraged its heritage by creating cultural merchandise and producing popular TV series such as “National Treasure” and “The Story of the Palace Museum,” fostering a strong brand identity. Another example is that, in the aftermath of the COVID-19 pandemic, some Chinese cities, such as Wuxi and Yangzhou, have implemented AR and VR technologies to provide “virtual nightscapes” to facilitate online tourism experiences. This provides new perspectives and business approaches for utilising tourism resources and providing new experiences to tourists. We have added the above information to Section 5.2. Developing sustainable tourism through comprehensive transportation systems and tourism resource optimisation in the revised manuscript.

**References**

Van Donkelaar, A., Hammer, M. S., Bindle, L., Brauer, M., Brook, J. R., Garay, M. J., ... & Martin, R. V. (2021). Monthly global estimates of fine particulate matter and their uncertainty. *Environmental Science & Technology*, *55*(22), 15287-15300.

https://pubs.acs.org/doi/10.1021/acs.est.1c05309

Sabo, F., Corbane, C., Florczyk, A. J., Ferri, S., Pesaresi, M., & Kemper, T. (2018). Comparison of built‐up area maps produced within the global human settlement framework. *Transactions in GIS*, *22*(6), 1406-1436.

Shah, M. I., Khan, Z., Moise, M. L., & Abbas, S. (2022). Tourism adaptability amid the climate change and air pollution in BRICS: a method of moments quantile regression approach. *Environmental Science and Pollution Research*, *29*(57), 86744-86758.

Gong, P., Li, X., Wang, J., Bai, Y., Chen, B., Hu, T., ... & Zhou, Y. (2020). Annual maps of global artificial impervious area (GAIA) between 1985 and 2018. *Remote Sensing of Environment*, *236*, 111510.

Reviewer #2: - This is an interesting paper.
- Gap and Contribution: The paper needs to improve the indentification of the gap and its contribution. The arguement used for a cotrasting view is based on two different context of papers. One is based on people's perception and the other uses secondary data. Therefore to make the arguement, both contrasting views should be based on the same type of data.

**[Our response]** Thank you very much for your advice. We have revised Section 1. Introduction in the revised manuscript to address those issues.

- Policy implications based on findings: The findings are interesting and therefore the author(s) need to provide appropriate and specific policy implications based on this research.

**[Our response]** Thank you very much for the suggestion. Yes, we have enriched the policy implications in our study. Accordingly, we have significantly revised Section 5. Discussions and policy implications in the revised manuscript.

- Please follow the journal’s formatting guidelines.

**[Our response]** Yes, sure! Thanks a lot!
