# Supplementary material for: How far can air pollution affect tourism in China? Evidence from panel unconditional quantile regressions
Source: PLoS One. 2024 Jun 7;19(6):e0304315. doi: 10.1371/journal.pone.0304315 (PMC11161063; doi:10.1371/journal.pone.0304315)
Supplement: Attachment — Submitted filename: Response to Reviewer.docx [file pone.0304315.s003.docx]

Reviewer #1: How Much Can Air Pollution Influence Tourism? Examination of 284 Chinese Prefecture-level Cities

Thanks for responding to my quieries – I am generally satisfied, just have couple of points to address before it cn make it to press:

1. Authors Response: Thank you for the question. Yes, factors such as weather conditions,

wind speed, and Air Quality Index (AQI) could potentially influence our findings.

These factors have been shown in prior studies to impact air pollution and tourism

development in individual cities or smaller samples. Regrettably, acquiring such

granular data is difficult in large-scale studies. Hence, we haven’t incorporated these

factors into this study.

Reviewer’s Response: Thank you for your response. I appreciate your acknowledgment of the potential influence of external factors like weather conditions, wind speed, and Air Quality Index (AQI) on your study's findings. It's understandable that acquiring granular data for such variables can be challenging in large-scale studies. Your decision to exclude these factors seems reasonable given the logistical difficulties in obtaining this detailed information.

However, it might be beneficial to discuss these limitations in your paper. Addressing the absence of these variables could provide readers with a clearer understanding of the study's scope and the context in which your findings are situated. This would also help in setting future research directions where such data might be more readily available or in smaller scale studies where the impact of these factors can be more feasibly assessed.

**[Our response]** Thank you very much for your suggestions. To address this issue, we have just acquired pertinent climate-related GIS data that assists in controlling the effect of climate conditions, such as precipitation, temperature, and wind speed, on tourism in our statistical models (see Section 3.1.3. Control variables). This dataset, procured from The National Tibetan Plateau Data Center (TPDC)’s China meteorological forcing dataset, encompasses annual observations for various cities from 2008 to 2018 with an impressive 1 km resolution (<https://data.tpdc.ac.cn/zh-hans/data/8028b944-daaa-4511-8769-965612652c49/>). While these variables have been duly incorporated as control variables into our statistical models, there are no significant changes in our statistical results, which attests to the robustness and reliability of our findings. Furthermore, the AQI data was obtained from a network of ground observation stations across China, constituting part of the official pollution monitoring data published by Chinese authorities. Our employed air pollution data have been calibrated in consideration of these ground observations (Hammer et al., 2020).

We provide the annual mean values of climate variables, which show minimal variation and thus have limited significance in the model. Besides, we have considered incorporating these climate variables as fixed effects; however, besides temperature, the inclusion of other variables seemed largely unnecessary. Yet, they are still included in the model. Additionally, to address the concern of multicollinearity, a VIF test is performed after these climate variables are added to the model. The mean VIF value of 1.61 indicates that multicollinearity is not a significant concern.

Reference:

Hammer, M. S., van Donkelaar, A., Li, C., Lyapustin, A., Sayer, A. M., Hsu, N. C., ... & Martin, R. V. (2020). Global estimates and long-term trends of fine particulate matter concentrations (1998–2018). Environmental Science & Technology, 54(13), 7879-7890.

2. My other question is about your model itself, and I am sorry to bring this up during the second review, but it is important for the credibility of your findings:

In the context of examining the relationship between air pollution and tourism development across a substantial number of cities over a decade, I have some concerns and questions regarding the handling of potential non-stationarity and trend components in your data:

Accounting for Linear Trends: The data spans 284 cities over a period from 2008 to 2018, which is a substantial time frame. However, it appears that linear trends have not been explicitly accounted for in your model. Could you elaborate on how your model addresses or accommodates potential linear trends in both the tourism development and air pollution variables over this period?

Potential Non-Stationarity Issues: Given the longitudinal nature of your data, there is a possibility of non-stationarity within the panel dataset. Non-stationarity can lead to biased and inconsistent coefficient estimates. How do you ensure that the variables in your panel data are stationary, or how do you mitigate the effects of potential non-stationarity?

Implications of Omitting Trend Components: If linear trends are not adequately accounted for, this might lead to spurious regression problems. Can you discuss the potential implications of this omission on the interpretation of your results, particularly regarding the coefficients' magnitude and significance?

Strategies for Trend and Non-Stationarity Adjustment: Have you considered using detrending methods or incorporating a trend variable in your model? Additionally, are there other econometric techniques, such as first differencing or cointegration analysis, that you have employed or could employ to address these issues?

You'll likely need to revise several models. A practical starting point would be conducting unit root tests specifically designed for panel data. For instance, using Stata, you can perform the Levin-Lin-Chu (LLC) test, Im-Pesaran-Shin (IPS) test, and Fisher-type tests based on augmented Dickey-Fuller (ADF) tests. If you prefer EViews, it also supports tests like the LLC, IPS, and Maddala and Wu's Fisher-type tests for such analyses. Moreover, the R programming language offers robust packages like plm and urca for panel data analysis, which include unit root tests such as the LLC and IPS tests.

I recommend applying these unit root tests to all your OLS models. Ideally, we won't find any unit roots, as their presence could complicate matters, potentially requiring significant revisions. Even if the tests for unit roots are negative, consider including a trend variable in your models, possibly both linear and quadratic trends, to enhance the robustness of your analysis.

Good luck with revision

**[Our response]** Thank you very much for your comments. There are two types of panel data: short panels, where the dimension of individual entities surpasses that of time, and long panels, where the time dimension exceeds that of individual entities. For the short panels, linear trends tend to be less discernible. In reviewing the scholarly literature on China, the studies performing unit root tests to adjust for linear trends are those grounded on provincial data that spanned longer than twenty years (i.e., long panels).

Despite our data belonging to the short panels, we have implemented unit root tests in the revised manuscript to assuage concerns about this topic. Our data was analyzed using Stata in accordance with instructions provided on the accompanying webpage (<https://www.stata.com/manuals/xtxtunitroot.pdf>). We have employed the Im-Pesaran-Shin test (IPS) and the Harris-Tzavalis test (HT) for the panel unit root tests.

The IPS test serves as our primary tool for decision-making, with IPS testing imposing specific minimum requirements on the time dimension T. In models that only include panel-specific means, a strongly balanced dataset requires T to be at least 5; if time trends are also included, T must be at least 6. For unbalanced data, the minimum requirement for T is 9. Should these minimum T requirements not be met, the p-value for Z_t-tilde-bar will not be reported. Given that our dataset’s dimensions exceed these T thresholds, the IPS method is thus our primary testing approach.

In parallel, the Harris-Tzavalis test is also considered an appropriate unit root test. A key assumption of the HT test is that the number of time periods, T, is fixed. This contrasts with other tests that assume T tends to infinity. Baltagi (2008) notes that a fixed T is typical in micropanel studies. For example, it might be more natural to expand the sample size by collecting more firm-level data, even though the number of time periods for each firm remains fixed. According to Baltagi (2008), our dataset also meets the requirements for this test.

Although the Levin-Lin-Chu (LLC) test is the most commonly used unit root test, it is generally recommended for moderate-sized panels (between 10 and 250 individuals with 25 to 250 observations per individual). While our dataset has a larger number of individuals, the observations per individual fall short of the recommended minimum of 25. Other methods, such as Fisher-type tests, assume T tends to infinity, and the Hadri test is theoretically valid when T tends to infinity. The statistic in Breitung’s test has an asymptotically normal distribution when first T and then N tend to infinity.

We have stated the IPS and HT unit root test results in the revised manuscript (see Table 3), which is complemented by a detailed description in the main text (see Section 4.1. Unit root test). The test results revealed that all variables except GDP (ln*GDP*) and the percentage of the tertiary industry (ln*TER*) passed the tests and did not exhibit unit roots. Therefore, we took the first-order difference of ln*GDP* and ln*TER* for the subsequent statistical analysis.

Reference:

Baltagi, B.H. (2008). Econometric Analysis of Panel Data. 4th ed. New York: Wiley.
