# Supplementary material for: How far can air pollution affect tourism in China? Evidence from panel unconditional quantile regressions
Source: PLoS One. 2024 Jun 7;19(6):e0304315. doi: 10.1371/journal.pone.0304315 (PMC11161063; doi:10.1371/journal.pone.0304315)
Supplement: Attachment — Submitted filename: Response to Reviewer.docx [file pone.0304315.s004.docx]

Reviewer #1: Good work. But believe it's essential to include a trend component in the regression model. Despite the authors having conducted unit root tests, my previous comment underscored the importance of incorporating trend variables, possibly both linear and quadratic, to strengthen the robustness of your analysis. It would be advisable for the authors to present two sets of results: one that includes the trend component and another without it. We want to make sure that your coefficients do not change very much. I strongly believe that the authors should include a trend component in their model to derive the coefficient, which could provide insights into technological progress or total factor productivity, depending on their interpretation. Good luck :)

**[Our response]** Thank you for the comment and suggestion. In the revised manuscript, we have added results related to linear and non-linear trends in Section 4.4. We employ the UQR-based quantile regression framework and use median regression to elucidate these results (i.e., the median model is a special form of quantile regression, similar to the mean regression but more robust). Table 8 displays our findings, which show no significant quadratic term, indicating that air pollution has no non-linear effects on tourism revenue or visits. Interestingly, the linear trend has a significant positive effect on tourist visits, which shows that public concern about pollution during travel diminishes over time. Your suggestion assisted us in discovering this intriguing phenomenon, and we appreciate your guidance in the quantitative analyses used in this manuscript.
